# Adverse outcomes in SAR-CoV-2 (COVID-19) and SARS virus related pregnancies with probable vertical transmission

**DOI:** 10.5935/1518-0557.20200057

**Published:** 2020

**Authors:** Gulam Bahadur, Roy Homburg, Wai Yoong, Cheentan Singh, Mamta Bhat, Phalguni Kotabagi, Santanu Acharya, Judith Huirne, Pablo Alexis Doreski, Mariusz Łukaszuk, Asif Muneer

**Affiliations:** 1 Reproductive Medicine Unit, Obstetrics & Gynaecology Department, North Middlesex University Hospitals Trust, London UK.; 2 Homerton Fertility Centre, Homerton University Hospital. London, UK.; 3 Neonatal and Peadiatric Unit, North Middlesex University London. London, UK.; 4 Ayrshire Fertility Unit, University Hospital Crosshouse, Kilmarnock, Scotland.; 5 University Medical Centers Amsterdam, Research Institute Reproduction and Development. Amsterdam, The Netherlands.; 6 Fundacion Respirar, Buenos Aires. Argentina; 7 Nowe Orlowo Medical Center, Gdynia, Poland; 8 University College London Hospital NIHR Biomedical Research Centre. London, UK.

**Keywords:** COVID-19, SARS-CoV-2, pregnancy, risks, vertical transmission

## Abstract

The global severe acute respiratory syndrome-related coronavirus SARS-CoV-2 (COVID-19) pandemic has had an unprecedented impact on all aspects of daily life and healthcare. Information on the infection risks for pregnant women and their offspring have so far been limited to small case series, until a large UK report on 427 SARS-CoV-2 infected pregnant women was published. Previous SARS epidemic experiences were drawn upon. Diagnostic use of real time polymerase chain reaction (RT-PCR) and IgG and IgM antibody tests are fraught with concerns of non-validation and false negative results, as are sampling methodologies. Virtually no information on controls accompany these reports. Infection of the mother and baby has serious implications for obstetric and neonatal care. Information on early and late stage pregnancy infection and the relationship to severity of infection on fetal development is both useful and clearly warranted. An increasing number of reports centre around mildly infected women showing no evidence of fetal infection while a few reports suggesting vertical transmission require further validation. Vertical transmission from mother to baby however small would have profound health implications for obstetric and neonatal care and fetal abnormalities. Some data suggesting intrapartum vertical transmission from mother to baby cannot be dismissed given the lack of controls and limitations of diagnostic viral tests. This analysis covers some key early reports addressing pregnancy outcomes following SARS-CoV-2 infection.

## Background

The global SARS-CoV-2 (COVID-19) pandemic has focussed unprecedented public health priorities and suspended all fertility treatment to prevent the spread of infection. Previous SARS pregnancies were associated with a high incidence of spontaneous preterm delivery, miscarriage, and intrauterine growth restriction, but provided no evidence of perinatal SARS infection amongst the infants who were born (Wong *et al.*, 2004). The data collections for the various periods were heterogeneous with globally localised experiences such as USA. Even with the current SARS-CoV-2 pandemic, there is no centralised linked data collection for pregnancy and neonatal outcomes. In the UK this role should be covered by the Office of National Statistics (ONS, 2020), and by an independent research group UK Obstetric Surveillance System (UKOSS). Submitting data to professional societies or individual research groups is voluntary and prone to bias especially if permission from hospitals is restricted with small numbers, potentially breaching privacy, or withheld to prevent unnecessary media attention. Of particular interest to clinicians is it vertical transmission from mother to baby occurs.

Angiotensin-converting enzyme 2 (ACE2) is the receptor for SARS-CoV-2, and has a central role in human infection and ongoing transmission. ACE2 is highly expressed in maternal-fetal interface cells which includes stromal cells and perivascular cells of decidua and cytotrophoblast as well as syncytiotrophoblast within the placenta. Therefore, SARS-CoV-2 associated vertical transmission and the placenta dysfunction/abortion need to be investigated in clinical practice (Li *et al.*, 2020). Preliminary unverified reports state that embryos harbouring SARS-CoV-2 receptors ACE2 and BSG (CD147), the serine protease TMPRSS2 and the endosomal protease CTSL also raise concerns about the possible hazard to the developing embryos and hence early pregnancy loss or complications (Colaco *et al.*, 2020). ACE2 and TMPRSS2 expression in oocytes, ovarian tissue and testicular tissue serve as possible targets for SARS-CoV-2 virus (Stanley *et al*.,2020). Therefore, IVF procedures present several levels of complexities over and above the known risks of surgical procedures, multiple births and OHSS. In contrast, IUI in the SARS-CoV-2 post pandemic provides exceptionally low risk, low intrusive benefits and a logistical fertility treatment pathway (Bahadur *et al*., 2020).

We highlight the key pregnancy related reports appearing up to 26/05/2020.

## Possible vertical transmission

From previous virology reports, the rate and risk of viral transmission appears to increase with advancing fetal gestation. Once viruses cross the placental barrier, depending on gestation, they can cause serious fetal effects including birth defects, abnormalities of growth and development, neurological injuries, miscarriage, fetal death, preterm delivery and neonatal complications (Silasi *et al.*, 2015; Schwartz & Schwartz, 2020). Vertical transmission mechanisms are poorly understood (Schwartz & Schwartz, 2020), and could possibly be through a maternal hematogenous route via the maternal-fetal interface. Viruses circulating in the maternal bloodstream enter the placenta from uterine arteries, circulate in the intervillous space, and can pass to the fetus through the chorionic villous tree where they eventually enter the fetal circulation (Schwartz & Schwartz, 2020).

Various reports exist for pregnancy and neonatal infections. Very early cases of neonatal infections provide significant information on the maternal and neonatal interrelationship. One study found that three neonates tested positive for SARS-CoV-2 and one neonate was infected with SARS-CoV-2 36h following delivery (Yu *et al.*, 2020). One neonate which was 16 hours post-delivery was found to be positive using real-time polymerase chain reaction (RT-PCR) but the immunoglobulin IgM and IgG for SARS-CoV-2 were both negative, suggesting a possible vertical transmission. Interestingly, there were no maternal antibodies detected until after delivery and all three babies had positive RT-PCR on day 2 (Alzamora *et al.*, 2020). The report does not include whether there was an antibody response in the neonate. Three neonates had elevated SARS-CoV-2 IgG and IgM levels but the RT-PCR test was negative. Two new-born babies delivered by Caesarean section tested positive for SARS-CoV-2 by RT-PCR assay which suggests vertical or peripartum transmission of SARS-CoV-2 (Parazzini *et al.*, 2020). The earliest single case report of a 2-hour newborn with elevated SARS-CoV-2 IgM antibodies to be born to an infected mother suggests that the neonate was infected in utero and points to possible vertical transmission (Dong *et al.*, 2020). The large macromolecular IgM unlike IgG is unlikely to cross the placenta from the maternal compartment to the fetus (Zeng *et al.*, 2020). RT-PCR testing of the patient’s vaginal secretions and later on the breast milk were negative. Potentially, the infant could have been exposed for 23 days from the time of the mother’s diagnosis of SARS-CoV-2 to delivery. IgM antibodies do not appear until 3 to 7 days post infection and are not transferred to the fetus via the placenta; therefore, infection was least likely during delivery. The infant’s repeatedly negative RT-PCR test results were difficult to explain and such tests are not always positive with infection (Dong *et al.*, 2020). Caution should still be exercised given the sensitivity and specificity of the IgM assay was 70.2% and 96.2% respectively. SARS-CoV-2 infection positive vertical transmission was described in one of the seven (14.3%) neonates tested within the first 24-36 hours of life (Hu *et al.*, 2020). The data suggested vertical transmission of SARS-CoV-2 infection from mothers during the last few days of pregnancy was possible but urge caution in the interpretation. Perinatal SARS-CoV-2 infection and vertical transmission is also hypothesized (Chen *et al.*, 2020a; Zeng *et al.*, 2020a). An early-onset neonatal infection with elevated IgM antibody to the virus could not confirm intrauterine vertical transmission of SARS-CoV-2 (Schwartz & Schwartz, 2020).

In a COVID-19 infected neonate case report showed positive RT-PCR assay 36 hours after birth, but had negative cord blood and placenta. There was insufficient information to establish vertical transmission from the mother to the child (Wong *et al.*, 2020). RT-PCR was positive in the amniotic fluid and throat swab at 24 hours, for the baby born at 32 weeks gestation to the mother with symptomatic COVID which strongly raises the possibility of vertical transmission (Zamaniyan *et al.*, 2020). Transplacental vertical transmission could not be excluded in 5 out of 70 cases (7.1% of neonates) (Fornari, 2020).

Newer cases for vertical transmission from mother to baby are recorded. In the first report of its type, 3/11 placental swabs tested positive for SARS-CoV-2 RNA although the babies remained negative (Penfield *et al.*, 2020). Despite the limitations of reporting a single case without RT-PCR of amniotic fluid or placenta one newborn did have elevated SARS-CoV-2 IgM antibodies to a mother infected with COVID-19 (Dong *et al.*, 2020). RT-PCR on amniotic fluid and infant tested positive for COVID-19 infection (Zamaniyan *et al*., 2020).

## Placental pathology after viral infection

If antepartum vertical transmission from mother to baby is to be confirmed then ideally SARS-CoV-2 virus detection by electron micrography to the placenta, cord blood and amniotic fluid should occur. The placenta is an immune- privileged site and viral detection is complex because of the various compartments. Confirmation of viral damage to placenta is another line of evidence or is isolating and incubating tissue to release the virus to check for infectivity. The challenges of identifying SARS-CoV-2 virus were highlighted in infected endothelial cells using electron microscopic and where ribosomes appeared to be identified rather than the spikes on coronavirus particles (Goldsmith *et al*., 2020).

Compared to historical controls, placenta from 16 patients infected with SARS-CoV-2 showed decidual arteriopathy and maternal vascular malperfusion (Shanes *et al.*, 2020). The SARS-CoV-2 infected placenta however, showed no significant increase in acute or chronic inflammatory pathology. Third trimester placentas showed at least one feature of maternal vascular malperfusion (MVM), particularly abnormal or injured maternal vessels, and intervillous thrombi. The placenta of one second trimester fetal demise had villous edema and a retroplacental hematoma (Shanes *et al*., 2020). One infected patient was hypertensive despite the association of MVM with hypertensive disorders and preeclampsia. These changes may reflect a systemic inflammatory or hypercoagulable state influencing placental physiology. Placental injury affects intervillous space oxygenation which in turn is associated with adverse perinatal outcomes (Shanes *et al*., 2020). None of the SARS-CoV-2 associated placentas were tested for viral RNA or protein. The abnormal maternal circulation and an increased incidence of chorangiosis gives a mechanistic clue into the infection related changes.

Similar morphological and anatomic-pathological changes were noted during Zika virus infections. These included; increased stromal cellularity, villitis, calcification, maternal vascular malperfusion, placental hypoplasia, and maternal-fetal haemorrhage (intervillous thrombi). Different regions of the placenta such as the umbilical cord, amniotic membrane, chorionic plate, chorionic villus, and basal plate from pregnant women can be infected with the Zika virus. However, the collection and storage of the placenta were vital in the successful detection of Zika (Venceslau *et al*., 2020). Following viral infection, endometrial microbiota changes alter endometrial receptivity, giving higher miscarriage rates and poor implantation rates following IVF treatments (Moreno *et al*., 2016).

Increased endometrial expression of ACE2 with gonadotropin-induced changes in animal models need further evaluation in human fertility treatments (Pereira *et al*., 2009). Equally, the increased expression of ACE2 in the glandular epithelium of mid-to-late secretory phase of the menstrual cycle (Vaz-Silva *et al*., 2009) raises the risk for localised SARS-CoV-2 infections.

Two reports make a compelling case for vertical transmis- sion showing SARS-CoV-2 viral particle and or damage to the placenta (Hosier *et al*., 2020, Kirtsman *et al*., 2020). Electron microscopy of SARS-CoV-2 particles within the chorionic villi and in different placental cell types, cytotrophoblast, syncytiotrophoblast and fibroblast showed SARS-CoV-2 was found mainly in the syncytiotrophoblast cells at the maternal-fetal interface of the placenta in a second trimester of pregnancy (Hosier *et al*., 2020). This is probably the first case of transplacental transmission and reinforces the concern and risk with infectious disease in neonates. Congenital SARS-CoV-2 infection, with virus present in a neonate’s nasopharynx at the time of birth suggest vertical transmission may have occurred (Kirtsman *et al*., 2020). The placental photomicrographs showed broad zones of cellular infiltrates with infiltration of the intervillous space by chronic inflammatory cells. The chorionic villi showed extensive early necrosis of the syncytiotrophoblast layer and there was early infarction, with extensive smudginess and early fragmentation of the syncytiotrophoblast nuclei (Kirtsman *et al*., 2020).

## The evidence for no vertical transmission

Most cases exclude vertical transmission. No evidence of vertical transmission was identified in a cohort of 10 babies born to 9 mothers, all having negative throat swabs for SARS-Cov-2 using RT-PCR taken between day 1 to day 9 (Zhu *et al.*, 2020). Furthermore, no vertical transmission of COVID-19 was found in the third trimester of pregnancy amongst neonates delivered vaginally (Khan *et al.*, 2020; Xiong *et al.*, 2020). In 31 COVID-19 infected pregnant mothers, no COVID-19 infection was detected in neonates or in the placenta. Two mothers died from COVID-19-related respiratory complications postpartum but there was no evidence for intrauterine transmission of COVID-19 from infected pregnant women to the fetus. An increased risk for severe respiratory complications for infected mothers is reported (Karimi-Zarchi *et al.*, 2020). Equally, an analysis from 55 COVID-19 infected pregnant women and 46 neonates suggests that there is no definite evidence of vertical transmission (Dashraath *et al.*, 2020). In women diagnosed with SARS-CoV-2 in the 3rd trimester, no definitive evidence of intrauterine vertical transmission during pregnancy was demonstrated, whilst acknowledging transmission in the 1st and 2nd trimester remains unknown and that high-quality analyses would be required to rule out vertical transmission (Cheruiyot *et al.*, 2020).

It may not be surprising that vertical transmission is not found in mildly infected pregnant women or if there is a very short duration of maternal infection exposure to the early stage of fetal development. SARS-CoV-2 virus crossing the placental barrier needs to be confirmed especially in severely infected mothers (Kimberlin & Stagno, 2020). Interpreting negative RT-PCR along with numerous sampling methods has severe limitations especially given there is a latent incubation period for the virus. In SARS-CoV-2 infected adults, most individuals tested negative and only became positive after repeated testing on days which followed (Kelly *et al*., 2020). The period from symptoms occurring to RT-PCR test becoming positive presents high risk for spreading the virus. We therefore do not have a full understanding as to when the optimal sample should be taken. Researchers are suggesting samples taken at the maternal and neonatal interphase should be retained for later research, presumably when testing improves.

## UK experience on SARS-Cov-2 infection in pregnant women

In the UK, UKOSS, reported on 427 pregnant women diagnosed with SARS-CoV-2 the pregnant women were no more likely than non-pregnant women to become severely ill with SARS-Cov-2 (Knight *et al.*, 2020). However, there was a greater likelihood of becoming severely ill in their 3rd trimester of pregnancy. Co-morbidities were deemed to be important factors; overweight, older pregnant women or pregnant women with pre-existing comorbidities, such as hypertension and diabetes, were more likely to be admitted to hospital with SARS-Cov-2. Black and ethnic minority pregnant women were also more likely to be admitted to hospital with SARS-Cov-2 infection. Of these, 1 in 5 babies were born prematurely and required admission to a neonatal unit, fewer than 20 babies were born very premature. One in 20 babies born tested positive for SARS-Cov-2, but only half of these babies had a positive test immediately after birth. Whilst this study is suggesting low transmission of infection from mother to baby, the science and limitations of the diagnostic tests where both validation and uncertainty measurements remain absent, the true size of infected neonates remains to be seen (Knight *et al.*, 2020). UKOSS revealed 247 women (58%) gave birth or had a pregnancy loss; 180 (73%) gave birth at term, 40 (9%) hospitalised women required respiratory support. Of the 12 infants (5%) testing positive for SARS-CoV-2 RNA, 6 infants were positive within the first 12 hours after birth. Overall, 5 women admitted for SARS-CoV-2 died, a case fatality of 1.2% (95% CI 0.4-2.7%) and a SARS-CoV-2-associated maternal mortality rate of 5.6 (95%CI 1.8-13.1) per 100,000 maternities (Knight *et al.*, 2020). In contrast, the maternal mortality rate from H1N1 influenza was 1.6 per 100,000 pregnant women (Yates *et al*., 2010), although caution should be exercised in comparing heterogeneous and small sized cohorts. There remain major disparities in data collection from UK research interest groups and the main UK government ONS body gathering data. A Freedom of information request from the ONS revealed only 1 maternal death (ONS, 2020), whereas 5 deaths were reported through voluntary submission to UKOSS research group. This data would not capture the general population unreported deaths. Data collection in future pandemics will need better joined up structures and rules.

## Other pregnancy experience

In one systematic review of mothers infected with a variety of coronavirus infections that have caused epidemics in the recent past, 19 studies were included with 79 women: 41 pregnancies (51.9%) affected by COVID-19, 12 (15.2%) by MERS, and 26 (32.9%) by SARS. For all CoV infections, the rates of adverse effects were as follows: miscarriage (39.1%), preterm birth <37 weeks (24.3%), premature pre-labour rupture of membranes (20.7%), preeclampsia (16.2%), and fetal growth restriction (11.7%); 84% were delivered by Caesarean section; the rate of perinatal death was 11.1% and 57.2% for newborns admitted to the neonatal ICU. When focusing on COVID-19, the commonest adverse pregnancy outcome was premature birth <37 weeks, which occurred in 41.1% of cases, whilst the rate of perinatal death was quoted as 7% but none of the new-borns had vertical transmission (Di Mascio *et al.*, 2020). Fetal distress, premature labour, respiratory distress, thrombocytopenia accompanied by abnormal liver function and possibly death may be associated with SARS-COV-2 infection (Zhu *et al.*, 2020).

In another systematic review of 108 COVID-19 infected pregnancies, severe maternal morbidity and perinatal deaths were reported, whilst COVID-19 vertical transmission could not be excluded (Zaigham & Andersson, 2020). Most cases were in the 3rd trimester and associated with fever (68%), coughing (34%), lymphocytopenia (59%) with elevated C-reactive protein (70%) and 91% were delivered by caesarean section. There were no maternal deaths, but 3 maternal ICU admissions were required. One intrauterine death and one neonatal death was recorded. In neonatal blood sera from mothers infected 

with SARS-CoV-2, IgG concentrations in 5 infants were elevated. IgG usually transfers across the placenta from mother to fetus at the end of the 2nd trimester and reaches peak levels by the time of birth. On the other hand, IgM, was detected in 2 infants. IgM is not usually transferred from mother to fetus due to the large macromolecular structure (Zeng *et al.*, 2020b). The placenta of 2 SARS-CoV infected women in the third trimester of pregnancy had abnormal weights and pathology.

One experience suggests that there is no evidence of higher maternal or fetal risks and that pregnant women infected with SARS-CoV-2 would have similar outcomes to those non-infected pregnant women (Monteleone *et al.*, 2020). From, 18 studies comprising 114 mildly infected, 3rd trimester pregnant women, the fetal and neonatal outcomes appeared normal and without evidence of intrauterine vertical transmission (Yang *et al.*, 2020). In contrast, the first peer reviewed report detailed the death of a 27-year-old pregnant woman (Karami *et al.*, 2020). A second trimester miscarriage case related to SARS-CoV-2 placental infection was reported (Baud *et al.*, 2020). Miscarriage or fetal growth restriction was observed in 40% of maternal infections with MERS and SARS viruses (Favre *et al.*, 2020; Wong *et al.*, 2004) and there are insufficient cases to determine with confidence whether SARS-CoV-2 can cause similar adverse outcomes.

## Perspectives on the severity of infection and other viruses

What we do not yet know is how the severity of COVID-19 will impact on pregnancy (Dotters-Katz & Hughes, 2020). In 8% of the 118 COVID-19 positive pregnant women, severe infection developed and the severity of disease increased in 6 women following delivery (Chen *et al.*, 2020b). The majority of studies suggest careful monitoring of COVID-19 pregnancies with measures to prevent neonatal infection. There is a paradigm shift in the management of pregnant women during a pandemic whilst maintaining safety of patients and healthcare providers. Limited data on pregnant infected women suggests COVID-19 can have an association with higher risk of fetal respiratory distress and lung diseases. It would be beneficial to start analysing data comparing healthy pregnant women with women who have comorbidities and smoking status. The context of SARS-CoV-2 related pregnancies should be placed against Parvovirus B19, CMV, Rubella and VZV risks for which we have significant evidence base. The American Society for Reproductive Medicine (ASRM Task force) raises concerns regarding the usage and storage of gametes during this COVID-19 pandemic (Segars *et al*., 2020). Given the numbers of unknown asymptomatic pregnant women presenting in maternity units, clearly a high degree of clinical vigilance is required (Govind *et al*., 2020). On the other hand, where universal testing was undertaken in a cohort of 215 pregnant women, this revealed 13.5% who were asymptomatic, tested positive for RT-PCR SARS-CoV-2 (Sutton *et al*., 2020).

Interpreting virology test results remain overlooked in clinical and research settings (Sethuraman *et al*., 2020; Wölfel *et al*., 2020) while little attention has been made to factor in the humoral responses to SARS-CoV-2 (Cuo *et al*., 2020) as has been the incubation of the SARS-CoV-2 virus (Lauer *et al.*, 2020; Zhao *et al*., 2020). Perhaps most striking is to note that the false negative RT-PCR is 100% on day 1 of the infectious period, declining to 67% on day 4 post symptoms onset (Kucirka *et al*., 2020), it is remarkable so many negative single RT-PCR reports dominate the field and especially on the non-peer reviewed MerRxiv platform without any controls now being reported in the media. Furthermore, numerous reports are now incorporated in a plethora of systemic analyses, sometimes duplicating the cases when published elsewhere. So many SARS-CoV-2 positive babies under 12 -24 hours are now known. Placing into context when RT-PCR becomes effectively positive and the incubation period, then there is a strong likelihood that the SARS-CoV-2 neonatal infection pre-existed the birth, and more careful and controlled studies will be required in future. Reports which reassure that is no SARS-CoV-2 viral presence in semen appear misleading as RT-PCR tests performed after a median of 31 days (interquartile range, 29-36 days) post infection (Pan *et al*., 2020; Eisenberg ML, 2020) are likely to be negative anyway. The optimal RT-PCR testing is within the 6-12day period post infection (Guo *et al*., 2020; Kucirka *et al.*, 2020; Lauer *et al*., 2020; Sethuraman *et al*., 2020).

## Conclusion

Numerous reports suggest vertical transmission as a possibility and there is a likelihood of the infection being acquired peripartum. Factoring in SARS-CoV-2 virus incubation and diagnostics of when RT-PCR becomes positive may support vertical transmission in infected babies. However, more controlled data is required to decipher the underlying risks of maternal and fetal infections, possible vertical transmission and early pregnancy risk. Early pregnancy risks due to denuded oocytes and biopsied embryos need to be considered in IVF procedures given their potential susceptibility to SARS-CoV-2 infection. IUI provides a safer logistical early treatment option compared with multi-level complexities of IVF, offering reduced risks and non-surgical interventions. Maternal mortality rate of 5.6 (95% CI 1.8-13.1) per 100,000 maternities or a case fatality of 1.2% (95% CI 0.4-2.7%) in the UK is recorded. It appears around 10% of SARS-CoV-2 infected pregnant women require hospitalisation with respiratory support, while 5% (n=12) of infants tested positive for SARS-CoV-2 RNA, and half of these within 12 hours after birth. Co-morbidities are a major factor in SARS-CoV-2 infected pregnant women. Miscarriages, premature deliveries in the third trimester and neonatal infections are associated with SARS-CoV-2 infected pregnant women. As the science of diagnostics evolve information on optimal sampling and timing may confirm intrapartum vertical transmission from mother to baby which cannot be ruled out at this stage. Importantly, this limited information should be used to counsel and protect mothers and babies of the risks as well as protecting maternity and neonatal care teams from harm of SARSCoV-2 infection.
